# Exploring the Mechanisms and Preventive Strategies for the Progression from Idiopathic Pulmonary Fibrosis to Lung Cancer: Insights from Transcriptomics and Genetic Factors

**DOI:** 10.3390/biomedicines12102382

**Published:** 2024-10-18

**Authors:** Kai Xie, Xiaoyan Tan, Zhe Chen, Yu Yao, Jing Luo, Haitao Ma, Yu Feng, Wei Jiang

**Affiliations:** 1 Department of Thoracic and Cardiovascular Surgery, Medical Center of Soochow University, Suzhou 215000, China; kaixie715786420@126.com (K.X.); 20225256025@stu.suda.edu.cn (X.T.); czstudy1998@163.com (Z.C.); mhtszdx@163.com (H.M.); 2 Department of Respiratory Medicine, Nanjing University of Chinese Medicine, Nanjing 210000, China; yaoyuxixi1993@163.com; 3 Department of Cardiothoracic Surgery, Medical School of Nanjing University, Nanjing 210002, China; luojing_2767983@163.com; 4 Department of The First Clinical, Medical College of Soochow University, Suzhou 215006, China

**Keywords:** idiopathic pulmonary fibrosis, lung cancer, Mendelian randomization, WGCNA, SMR, *MS4A4A*

## Abstract

**Background:** Idiopathic pulmonary fibrosis (IPF) leads to excessive fibrous tissue in the lungs, increasing the risk of lung cancer (LC) due to heightened fibroblast activity. Advances in nucleotide point mutation studies offer insights into fibrosis-to-cancer transitions. **Methods:** A two-sample Mendelian randomization (TSMR) approach was used to explore the causal relationship between IPF and LC. A weighted gene co-expression network analysis (WGCNA) identified shared gene modules related to immunogenic cell death (ICD) from transcriptomic datasets. Machine learning selected key genes, and a multi-layer perceptron (MLP) model was developed for IPF prediction and diagnosis. SMR and PheWAS were used to assess the expression of key genes concerning IPF risk. The impact of core genes on immune cells in the IPF microenvironment was explored, and in vivo experiments were conducted to examine the progression from IPF to LC. **Results:** The TSMR approach indicated a genetic predisposition for IPF progressing to LC. The predictive model, which includes eight ICD key genes, demonstrated a strong predictive capability (AUC = 0.839). The SMR analysis revealed that the elevated expression of *MS4A4A* was associated with an increased risk of IPF (OR = 1.275, 95% CI: 1.029–1.579; *p* = 0.026). The PheWAS did not identify any significant traits linked to *MS4A4A* expression. The rs9265808 locus in *MS4A4A* was identified as a susceptibility site for the progression of IPF to LC, with mutations potentially reprogramming lung neutrophils and increasing the LC risk. In vivo studies suggested *MS4A4A* as a promising therapeutic target. **Conclusions:** A causal link between IPF and LC was established, an effective prediction model was developed, and *MS4A4A* was highlighted as a therapeutic target to prevent IPF from progressing to LC.

## 1. Introduction

Idiopathic pulmonary fibrosis (IPF) is a chronic and progressive lung disease characterized by the excessive deposition of fibrous connective tissue within the lungs, leading to a gradual loss of lung function and clinical manifestations such as difficulty breathing [1,2]. Fibroblasts play a crucial role in IPF, in which their persistent activation and proliferation result in the excessive accumulation of extracellular matrix components like collagen, contributing to the fibrotic remodeling of lung tissue [3]. This fibrotic environment shares several characteristics with tumor stroma, including altered tissue architecture and a pro-inflammatory milieu, which are conducive to the progression of potential cancer cells [4]. The incidence of lung cancer (LC) development is significantly higher in patients with IPF compared to those without IPF [5,6]. While the link between IPF and LC has been established, further research is needed to elucidate the causal foundations of this association.

Immunogenic cell death (ICD) is a form of cell death that incites an immune response against antigens from dead cells [7]. In IPF, the ongoing injury and death of lung epithelial cells can initiate ICD, leading to the release of danger-associated molecular patterns (DAMPs) and other damage signals [8]. These signals have the potential to create a pro-inflammatory environment within lung tissue. Chronic inflammation is a known risk factor for cancer, as it can cause DNA damage, promote cellular proliferation, and sustain tumor survival by fostering an immunosuppressive microenvironment [9,10]. Furthermore, persistent ICD and the accompanying inflammatory response in IPF may aid in the remodeling of lung tissue, establishing favorable conditions for oncogenic transformations. This continuous cycle of ICD in pulmonary fibrosis can thus contribute to a milieu that supports tumor initiation and progression, thereby linking IPF to a heightened risk of LC.

With the widespread adoption of whole-genome sequencing technologies and the enhancement of genomic databases, advanced methodologies like Mendelian randomization (MR) [11], Genome-Wide Association Studies (GWAS) [12], colocalization analysis, and Phenome-Wide Association Studies (PheWAS) [13] are increasingly employed to explore disease associations at the nucleotide level. These approaches offer valuable mechanistic insights into complex diseases, including IPF and its connection to LC. By enabling the identification of genetic variants that may underlie disease processes, these methods support the development of new clinical interventions and therapeutic strategies. Such advancements not only enhance our understanding of the genetic basis of disease but also pave the way for personalized medicine in which treatments can be tailored based on an individual’s unique genetic profile.

This study used TSMR analysis to uncover a causal relationship between IPF and LC. Through the application of the WGCNA R package, modules most closely associated with adverse progression were identified in LC and IPF datasets (GSE5388, GSE110147, GSE213001, GSE28042, GSE33566, and GSE93606). Genes within these highly correlated modules were found to be related to ICD, which led to the identification of common feature genes through cross-referencing. To further refine the selection of feature genes, four machine-learning algorithms were employed: Boruta, random forest, XGBoost, and LASSO. Subsequently, a prediction model based on the cross-referenced feature genes was developed using a multi-layer perceptron (MLP). A clinical diagnosis nomogram for IPF was constructed to evaluate its accuracy, and the model’s performance was predicted using single-cell datasets. SMR and PheWAS analyses were conducted to assess whether the expression of *MS4A4A* is causally related to IPF risk and safety. The expression of *MS4A4A* in neutrophils was associated with the progression of IPF to LC. Moreover, in vivo experiments confirmed that *MS4A4A* is a potential therapeutic target for preventing the progression of IPF to LC.

## 2. Materials and Methods

### 2.1. Data Collection and Processing

The GWAS data for idiopathic pulmonary fibrosis (IPF) were derived from two large population-based studies: the Global Biobank Meta-Analysis Initiative (GBMI) and the International IPF Genetics Consortium (IIGC). Meanwhile, the GWAS data for lung cancer (LC) were obtained from the FinnGen Research Project, version R11. Transcriptome datasets for IPF were downloaded from the GEO database (https://www.ncbi.nlm.nih.gov/, accessed on 10 April 2024) and included the GSE5388, GSE110147, GSE213001, GSE28042, GSE33566, and GSE93606 series. The LC transcriptome datasets were obtained from the TCGA database. The single-cell transcriptome dataset for IPF, GSE122960, was also retrieved from the GEO database. Furthermore, a compilation of 34 genes associated with ICDs identified in prior research was systematically organized [14]. Data for the IPF GWAS were sourced from the International IPF Genetics Consortium [15]. Patient information from the GSE70867 dataset was utilized to assess the potential role of key molecules in the prognosis of IPF patients. PheWAS analysis was performed using data provided by AstraZeneca (https://www.azphewas.com/, accessed on 19 April 2024) [16,17].

### 2.2. Two-Sample Mendelian Randomization (TSMR) Analysis

The causal relationship between IPF and lung cancer was investigated using TSMR. Instrumental variables (IVs) were selected according to the following criteria: (1) effect allele frequency greater than 0.01; (2) significant loci strongly associated with the phenotype (*p* < 5 × 10^−8^); (3) SNPs with low linkage disequilibrium within 1000 kb (r^2^ < 0.001); (4) strong IVs with an F-statistic greater than 10 [18]. Mendelian randomization analyses were conducted using the inverse-variance-weighted (IVW) method, MR–Egger intercept test, maximum likelihood, weighted median, and corrected and modified likelihood-based Mendelian randomization (cML-MA), with directional testing employed to exclude potential reverse causation. The Q test was used to assess heterogeneity in MR analysis, and potential heterogeneity bias was controlled using the IVW method with a random-effects model, where a *p*-value less than 0.05 indicated statistically significant heterogeneity. The MR–Egger intercept test was applied to assess horizontal pleiotropy, with statistical significance indicated by a *p*-value less than 0.05. The cML-MA method was used to balance potential pleiotropy bias, applying a Bayesian data perturbation (BIC-DP) approach when the GOF test *p*-value was less than 0.05 and the Bayesian information criterion (BIC) method otherwise. IVW was regarded as the primary result when directional consistency was observed across all five methods. The MR analysis process strictly adhered to the previously published reporting guidelines for observational studies in epidemiology using MR [19].

### 2.3. Summary-Data-Based Mendelian Randomization Analysis (SMR)

Cis-eQTL data from eQTLGen were utilized as the exposure [20], and IPF GWAS data from the International IPF Genetics Consortium were used for the SMR analysis [15]. This analysis aimed to assess the causal relationship between key molecules and the risk of IPF. A heterogeneity in dependent instruments (HEIDI) test was used to distinguish whether the exposure and outcome were driven by the same causal variant. SMR software (version 1.3.3) with default parameters was used for these analyses. A *p*-value of less than 0.05 was considered the threshold for a significant causal association, while a HEIDI test *p*-value greater than 0.05 indicated that the association between gene expression and disease risk was not driven by linkage disequilibrium [21].

### 2.4. Kaplan–Meier (KM) Survival Analysis

Survival curves were generated utilizing the Kaplan–Meier method and subsequently analyzed using the log-rank test. A *p*-value less than 0.05 indicated a significant difference in median survival time between the compared groups. The optimal cutoff values for high and low gene expression in the survival analysis were determined using a surv_cutpoint package (version 0.4.9.).

### 2.5. PheWAS

Data provided by AstraZeneca were utilized to conduct the PheWAS analysis, aiming to explore gene pleiotropy and potential adverse effects of key molecules that were not detected in the SMR analysis. In the UK Biobank, exome-sequencing data from 470,000 participants were analyzed to identify significant associations between key molecules and thousands of phenotypes, employing the officially recommended threshold of 1 × 10^−8^.

### 2.6. Weighted-Gene Coexpression Network Analysis (WGCNA)

Following the identification of thoracic respiratory diseases with a causal relationship to IPF, we further obtained DRGs (thresholds: for the LC dataset, *p* < 0.05, |LogFC| > 1; for the IPF dataset, *p* < 0.05, |LogFC| > 0.2), and expression levels were extracted from the amalgamated dataset for WGCNA [22]. Furthermore, the ICD-score was computed within the combined matrix utilizing ssGSEA [23]. We used the R package “WGCNA”, and, based on microarray data, we used conFigd with parameters set to power = 6, maxBlockSize = 5000, minModuleSize = 50, and mergeCutHeight = 0.25, aiming to pinpoint gene modules exhibiting the highest relevance to the ICD score. The process culminated in identifying the intersection of modules that showed the greatest correlation with the ICD score across various diseases.

### 2.7. Key Predictive Genes for IPF and the Development of a Predictive Model

To ascertain critical predictors for IPF, we utilized an ensemble of machine learning techniques, including Boruta feature selection [24], random forest (RF) [25], XGBoost (version 1.7.6) [26], and LASSO regression, aiming to identify pivotal factors by intersecting the outcomes derived from these methodologies. Next, a predictive model based on the genes identified at the intersection was constructed employing an MLP neural network [27].

### 2.8. Establishment and Assessment of a Nomogram

The integration of pulmonary fibrosis datasets, namely GSE5388, GSE110147, GSE213001, GSE28042, GSE33566, and GSE93606, resulted in a comprehensive dataset. This dataset was subsequently partitioned into training and testing subsets at a 7:3 ratio. Using the rms package alongside feature genes identified within the training subset, a feature map was constructed. The efficacy of the nomogram was independently verified against both the testing and training subsets. Calibration curves were used to evaluate the predictive accuracy of the nomogram model. Ultimately, the model’s clinical utility was ascertained via decision curve analysis (DCA) and examining the area under the curve (AUC) metrics.

### 2.9. scRNA-Seq Data Revealed Heterogeneity of Key Genes

Single-cell transcriptome datasets were acquired for four healthy individuals and four patients diagnosed with IPF disease from GSE122960. The Seurat package was used for the data analysis. The criteria for cell retention included a mitochondrial gene content less than 10% and the expression of genes within the range of 200 to 5000, with a minimum of three cells expressing each gene. To address sample-to-sample variability, the Harmony package was utilized to eliminate batch effects. Cell subpopulations were delineated using the “FindClusters” and “FindNeighbors” functions within Seurat [28], with subsequent visualization via UMAP. Gene expression within the model was illustrated employing UMAP.

### 2.10. Identification of IPF Subclasses

We applied consensus clustering [29] by utilizing a comprehensive collection of 2752 previously published metabolic genes [30] encompassing the entirety of known human metabolic enzymes and transporters. The primary goal was to categorize IPF samples into unique subtypes guided by gene expression profiles utilizing the Consensus ClusterPlus framework. This approach facilitated the differentiation of IPF samples into distinct subtypes. Following this stratification, we meticulously analyzed the immunological and metabolic distinctions present across the identified IPF subtypes, providing insights into their underlying biological heterogeneity.

### 2.11. Mouse Model

In this study, healthy male C57BL/6 mice, aged 6–8 weeks and with an average weight between 18 and 22 g, were used. A total of 12 mice from the Laboratory Animal Center of Soochow University were used. The mice were randomly assigned into four groups using a random number table method: the PBS group (n = 3), the BLM group (n = 3), the BLM-si-NC group (n = 3), and the BLM-si-*MS4A4A* group (n = 3). The mice were maintained under standard laboratory conditions with a 12 h dark/light cycle, at a temperature of 22 °C, and with a humidity level of 55% ± 2.5%. They were also provided with food and water. The cage was ventilated 70 times per hour and had a light/dark cycle of 12/12 h, with the lights turned on at 6:00 am. Following anesthesia, these subjects received a solitary intratracheal dose of bleomycin (BLM, 1.6 mg/kg, supplied by Selleck), which successfully established an experimental model of pulmonary fibrosis within a 7-day period. Post-induction, the four groups were subjected to intratracheal nebulization: the PBS and BLM groups received PBS, the BLM-si-NC group received an adenoviral vector containing si-NC, and the BLM-si-*MS4A4A* group received an adenoviral vector containing *MS4A4A*. After 21 days, the subjects were euthanized through intraperitoneal injection of anesthetics comprising ketamine (100 mg/kg) and xylazine (10 mg/kg) to facilitate the collection of lung tissue sections and serum specimens for the evaluation of pulmonary fibrosis. The criteria for euthanizing the mice included a weight loss exceeding 15% of their baseline weight that did not recover after 3 days of normal feeding and significant deterioration in physical condition, such as sunken eyes, impaired breathing, or noticeably poor fur condition. All experimental mice met the criteria for euthanasia, with no survivors. The care, weight monitoring, and observation of the physical conditions of the mice were conducted by two independent researchers who were blinded to the experimental groups. This study was approved by the Institutional Animal Care and Use Committee at Soochow University Medical Center (the Fourth Affiliated Hospital of Soochow University, Suzhou Dushu Lake Hospital) Laboratory Animal Center (No. 2023-242172).

### 2.12. Quantitative Real-Time RT-PCR (qRT-PCR)

Total RNA was extracted from the mouse lung tissues using TRIzol reagent (Invitrogen, Waltham, MA, USA). A NanoDrop 8000 (Thermo, Waltham, MA, USA) was used to detect the concentration and purity of the RNA by measuring the absorbance at 260 nm and the 260/280 absorbance ratio. A cDNA synthesis kit (Takara, Cat: RR036A, KeyGEN, Nanjing, China) was used for the reverse transcription of 1 μg of total RNA. qRT-PCR (using 0.5 μL of forward primer, 0.5 μL of reverse primer,10 μL of SYBR premix, 4 μL of cDNA, and 15 μL of RNase-free water) was performed by SYBR Green to detect the relative mRNA expression level. The sequences of the primer used were as follows: *ACTIN* forward, 5′-*GTCATTCCAAATATGAGATGCGT*-3′; *ACTIN* reverse, 5′-*GCATTACATAATTTACACGAAAGCA*-3′; *MS4A4A* forward, 5′-*CTGGGAAACATGGCTGTCATA*-3′; *MS4A4A* reverse, and 5′-*CTGGGAAACATGGCTGTCATA*-3′.

### 2.13. Immunohistochemistry (IHC)

Lung tissues from the mice subjected to the experimental treatments were collected and fixed in 4% paraformaldehyde (Solarbio, Beijing, China). The tissues were then embedded in paraffin blocks and sectioned. A sequential dehydration process was conducted using a series of xylene and graded ethanol solutions. After incubation with 3% hydrogen peroxide to quench endogenous peroxidase activity, the sections underwent antigen retrieval via heating in a citrate buffer and were allowed to cool to room temperature. The samples were blocked with 5% bovine serum albumin for 30 min. Subsequently, the membranes were incubated with a primary antibody overnight in a shaker at 4 °C. The following day, the sections were treated with a biotinylated secondary antibody, followed by amplification using a streptavidin–horseradish peroxidase complex. Visualization was achieved using 3,3′-diaminobenzidine as the chromogen (Sigma, Burlington, MA, USA). Immunohistochemical analysis was conducted using a fluorescence microscope.

### 2.14. Determination of Hydroxyproline

The collagen content in the mouse lung tissue was measured using a hydroxyproline assay kit (Jiancheng, Nanjing, China). According to the manufacturer’s instructions, 45 mg of mouse lung tissue was weighed and hydrolyzed in a test tube. After adjusting the pH, the supernatant was collected, and the absorbance was measured at 550 nm.

### 2.15. Statistical Analysis

Data from public databases were analyzed using R software (version 4.1.2). For data with a normal distribution, a Pearson correlation analysis was employed, whereas a Spearman correlation analysis was used for data with non-normal distributions or different dimensions. A Kruskal–Wallis rank sum test and Wilcoxon rank sum and signed-rank tests were performed to assess the significance of differences among multiple variables and between two variables, respectively. A Kaplan–Meier survival analysis was conducted utilizing the “survival” package, and survival differences between groups were compared using a log-rank test. Relative risks were described by calculating hazard ratios (HR) and 95% confidence intervals (CI). A “pROC” package was applied for ROC curve analysis to evaluate the performance of classification models by plotting ROC curves and computing the area under the curve (AUC), which assesses model accuracy through sensitivity and specificity at various thresholds. Hierarchical clustering was utilized to categorize samples, revealing underlying structures in the data. Prior to clustering, data normalization was conducted to ensure comparability across variables. Odds ratio (OR) tests were conducted to evaluate the relative probability of two events occurring, exploring the impact of various variables via a binary logistic regression model. Venn diagrams were used to display overlaps between two or more datasets, facilitating visual understanding of variable relationships. Throughout these analyses, the assumptions inherent to each test were verified: OR testing assumed linear relationships between events and variables, with independent samples; hierarchical clustering presumed reasonable distance measures and suitable clustering methods for data structure; ROC curve analysis assumed the classification model had adequate discriminative power with well-set thresholds; Venn diagrams assumed significant correlations with meaningful overlaps; KM survival analysis assumed independent survival time data with minimal influence from censored data. Experimental data were analyzed using GraphPad Prism 9. Data are presented as mean ± standard error (SE). Each group comprised independent samples. Statistical differences between two groups were evaluated using the independent samples *t*-test. Data normality was verified with the Shapiro–Wilk test, and homogeneity of variances was confirmed using Levene’s test, with all data passing these checks. *p* < 0.05 indicated a statistically significant difference.

## 3. Results

### 3.1. TSMR Analysis and Sensitivity Analysis of IPF and LC

**Figure 1:**
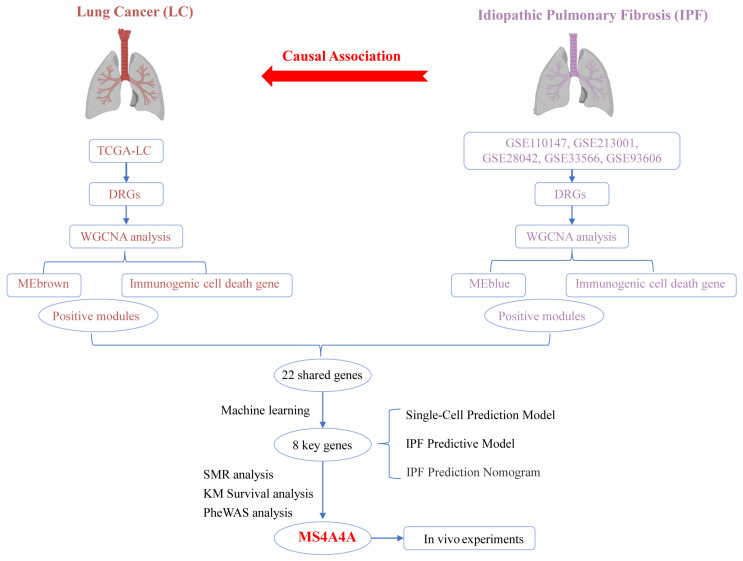
Overall study design and analysis flowchart.

Figure 1 shows the flowchart of the overall study.

**Figure 2:**
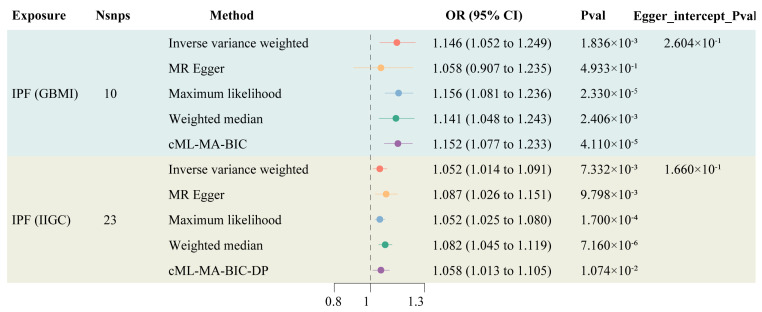
Forest plot of TSMR of IPF and LC.

Information on the instrumental variables derived from two genetic sources for IPF is presented in Supplementary Table S1, with F-statistics all exceeding 10, indicating a low likelihood of weak instrument bias affecting the results. The TSMR results indicated an increased risk of LC among IPF patients (OR: 1.146, 95% CI: 1.052–1.249). After controlling for horizontal pleiotropy, cML-MA demonstrated the same positive conclusion as the IVW method (OR: 1.058, 95% CI: 1.013–1.105) (Figure 2). The other methods maintain a consistent direction of causal effect with the above two methods. During the calculation of causal effects, horizontal pleiotropy tests and sensitivity analyses were conducted to ensure the accuracy and robustness of the results. The findings suggest that there is no statistically significant pleiotropy or reverse causation in the genetically predicted causal effect of IPF on LC (Supplementary Figures S1–S3).

### 3.2. Identification of ICD-Related Gene Modules in LC

**Figure 3:**
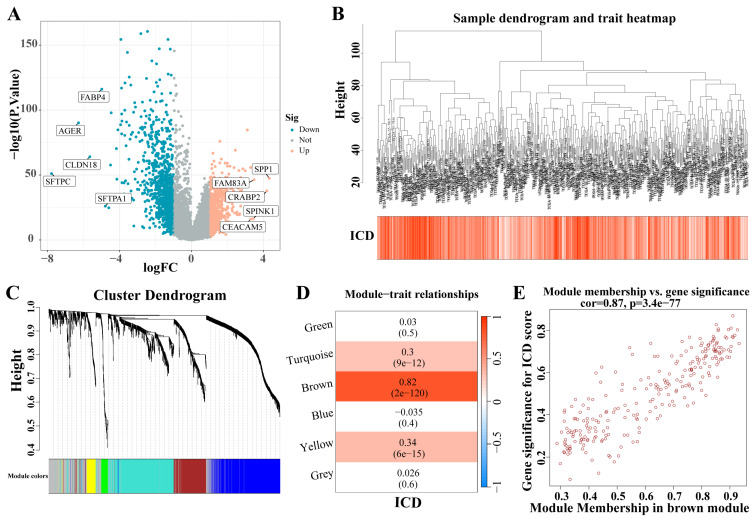
Identification of ICD-related gene modules in LC. (**A**) The volcano plot presents the expression pattern of DEGs in the LC dataset. (|LogFC| > 1, *p* < 0.05). Blue, downregulated genes. Yellow, up-regulated genes. (**B**) Sample dendrogram. (**C**) Clustering dendrogram of 1671 genes according to the measurement of dissimilarity. Genes are hierarchically divided into modules with different colors. (**D**) Heatmap of the correlation between module characteristic genes and LC; blue indicates negative correlation, and red indicates positive correlation. (**E**) Correlation of membership in MEbrown module with ICD-related genes. Abbreviations: LC, lung cancer; DEGs, differentially expressed genes; ICD, Immunogenic cell death.

A total of 1671 DEGs were identified from the analysis of the LC dataset (|LogFC| > 1, *p* < 0.05) (Figure 3A). These identified genes facilitated the construction of a co-expression network. The optimal soft threshold was determined to be a power of 6, ensuring the network’s scale-free topology. After excluding outlier samples, the co-expression network was successfully established (Figure 3B). Setting the minimum module size at 50 and the MEDissThres at 0.25 led to the identification of six distinct modules (Figure 3C). Notably, the MEbrown module exhibited a strong correlation with the ICD (Figure 3D). Furthermore, a significant correlation was demonstrated in the scatter plot of module membership (MM) (correlation = 0.87, *p* = 3.7 × 10^−77^; Figure 3E), indicating that the genes within the MEbrown module might possess functional relevance to the ICD.

### 3.3. Identification of ICD-Related Gene Modules in IPF

**Figure 4:**
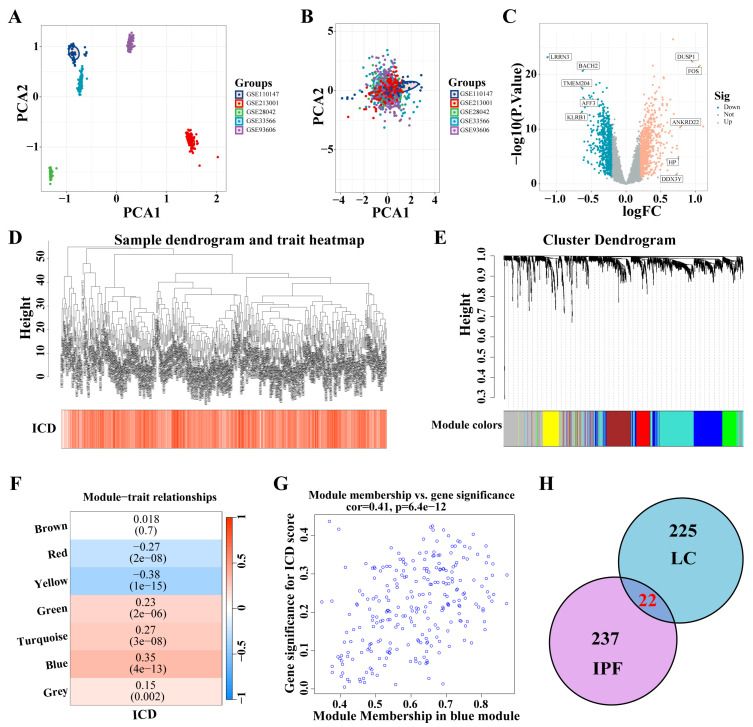
Identification of ICD-related gene modules in IPF. (**A**,**B**) PCA of five datasets before A and after B batch effect removal. (**C**) The volcano plot presents the expression pattern of DEGs in the IPF dataset. (|LogFC| > 0.2, *p* < 0.05). Blue, downregulated genes. Yellow, up-regulated genes. (**D**) Sample dendrogram. (**E**) Clustering dendrogram of 1466 genes according to the measurement of dissimilarity. Genes are hierarchically divided into modules with different colors. (**F**) Heatmap of the correlation between module characteristic genes and IPF; blue indicates negative correlation, and red indicates positive correlation. (**G**) Correlation of membership in MEblue module with ICD-related genes. (**H**) Venn diagram of crossing genes of ICD-related modules in IPF and LC. Abbreviations: IPF, Idiopathic pulmonary fibrosis; PCA, Principal component analysis; DEGs, differentially expressed genes; ICD, Immunogenic cell death.

Five IPF datasets were combined, and ComBat was utilized to adjust for batch effects (Figure 4A,B). A total of 1466 DEGs were identified from the analysis of the IPF datasets (|logFC| > 0.2, *p* < 0.05) (Figure 4C). These DEGs facilitated the construction of a co-expression network. The network’s optimal soft threshold was determined to be a power of 6, ensuring a scale-free network topology. Upon excluding outliers, the co-expression network was finalized (Figure 4D). Setting the minModuleSize to 50 and the MEDissThres to 0.25 led to the identification of seven distinct modules (Figure 4E). Notably, the MEblue module exhibited a pronounced correlation with the ICD (Figure 4F). The scatter plot for module membership (MM) revealed a significant correlation (correlation = 0.41, *p* < 6.4 × 10^−12^; Figure 4G), indicating the potential functional relevance of genes within the MEblue module to the ICD. A cross-analysis of ICD-related modules in LC and IPF revealed 22 shared genes (Figure 4H).

### 3.4. Selecting Key Genes and Constructing a Predictive Model

**Figure 5:**
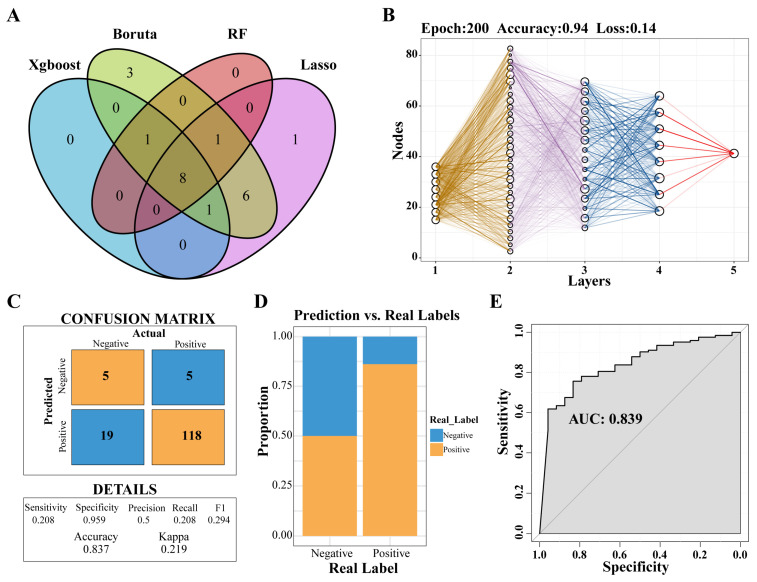
Selecting key genes and construction of predictive models. (**A**) Venn diagram showed the intersection of key genes obtained by the four algorithms. (**B**) Neural network analysis of key genes in IPF. (**C**) The prediction effect of the MLP classifier on the dataset of IPF. (**D**) The negative and positive ratio of the predicted model to the real label. (**E**) ROC curve of prediction model. Abbreviations: IPF, Idiopathic pulmonary fibrosis; MLP, multilayer perceptron; ROC, Receiver-operating characteristic.

Through the application of four distinct machine-learning methodologies, eight genes were identified: *PLBD1*, *ANKRD22*, *HLA-DPA1*, *HLA-DOA*, *MS4A4A*, *CARD16*, *CD163*, and *NLRC4* (Figure 5A). Subsequently, a predictive model was developed utilizing the MLP approach (Figure 5B). An analysis of the confusion matrix revealed an accuracy of 0.837 (Figure 5C), while a histogram illustrated the comparison between actual and predicted negative and positive cases (Figure 5D). Furthermore, the ROC curve yielded an AUC of 0.839, indicating that the predictive model had a high degree of accuracy (Figure 5E).

### 3.5. Constructing an IPF Prediction Nomogram and Assessing Its Accuracy

**Figure 6:**
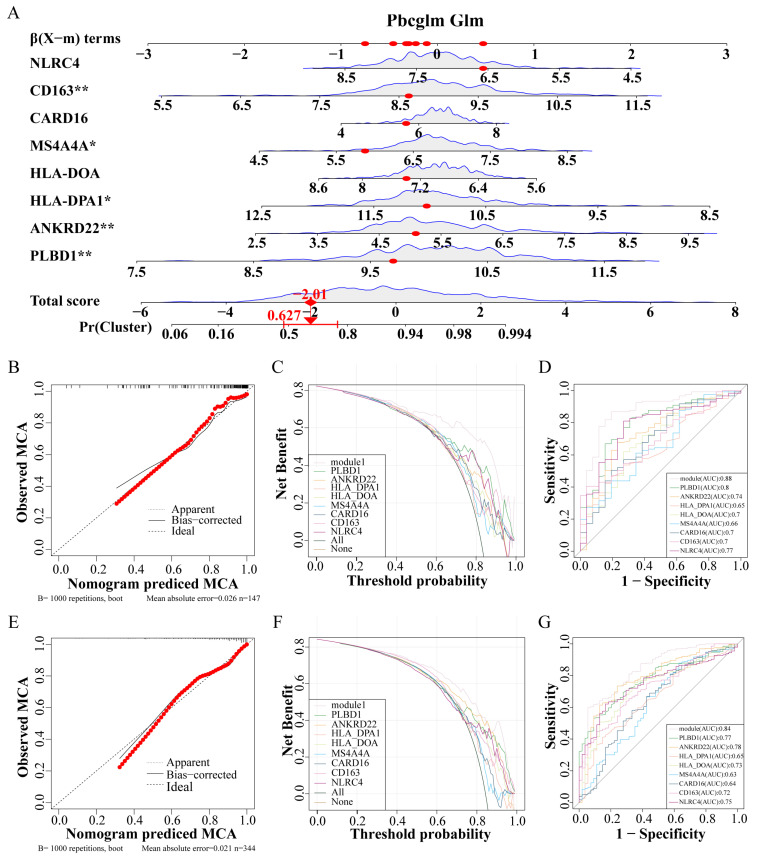
Construction and verification of IPF prediction Nomogram. (**A**) Nomogram model predicting incidence based on key genes. (**B**–**D**) The training set verifies the accuracy of the nomogram, calibration curve (**B**), decision curve (**C**), and ROC curve (**D**). (**E**–**G**) The test set verifies the accuracy of the nomogram, calibration curve (**E**), decision curve (**F**), and ROC curve (**G**). Abbreviations: IPF, Idiopathic pulmonary fibrosis; ROC, Receiver operating characteristic. * *p* < 0.05, and ** *p* < 0.01.

Utilizing these eight genes, a nomogram was developed for clinical diagnostic purposes (Figure 6A). The dataset was partitioned into a training set and a test set at a 7:3 ratio. In the training set, analyses including the calibration curve (Figure 6B), the decision curve (Figure 6C), and the ROC curve (Figure 6D) underscored the notable accuracy of the nomogram. Correspondingly, the test set calibration curve (Figure 6E), decision curve (Figure 6F), and ROC curve (Figure 6G) reaffirmed the diagnostic accuracy of the nomogram.

### 3.6. scRNA-Seq Data Revealed High Cellular Heterogeneity in Key Genes

**Figure 7:**
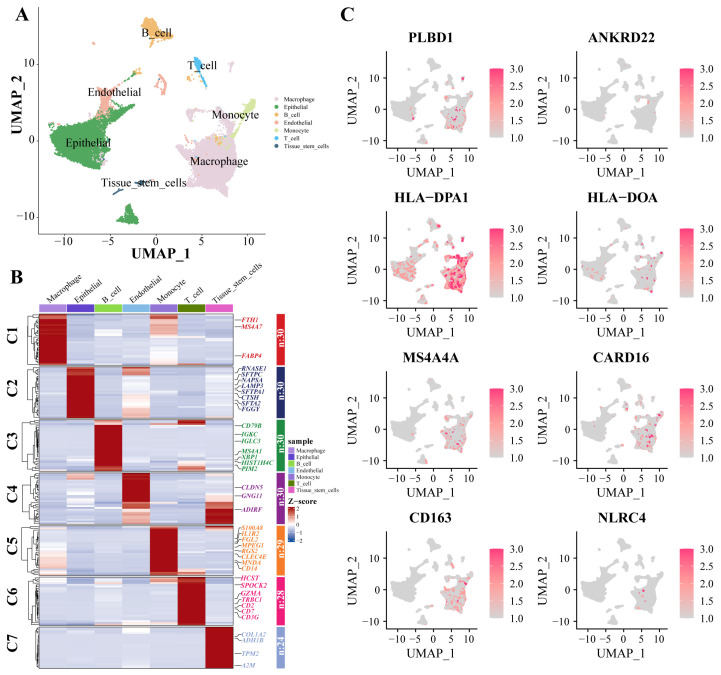
scRNA-Seq data show cellular heterogeneity of key genes. (**A**) IPF scRNA-seq analysis. Cluster analyzed by using UMAP method and populations were identified by color. (**B**) A heat map shows the hub genes in each sub-cluster. (**C**) The expression of eight key genes at the single-cell level. Abbreviations: IPF, Idiopathic pulmonary fibrosis; scRNA-seq, single cell RNA-sequence data; UMAP, uniform manifold approximation and projection.

Dimensionality reduction and annotation techniques were applied to the single-cell IPF dataset, resulting in the classification of IPF cells into seven distinct types: macrophage, epithelial, B_cell, endothelial, monocyte, T_cell, and tissue_stem_cells (Figure 7A). The genes were highly expressed within each cell type (Figure 7B). Furthermore, in the expression patterns of eight signature genes at the single-cell level (Figure 7C), all eight key genes were significantly expressed in macrophages.

### 3.7. ICD Subtypes and Associated Immune Characteristics

**Figure 8:**
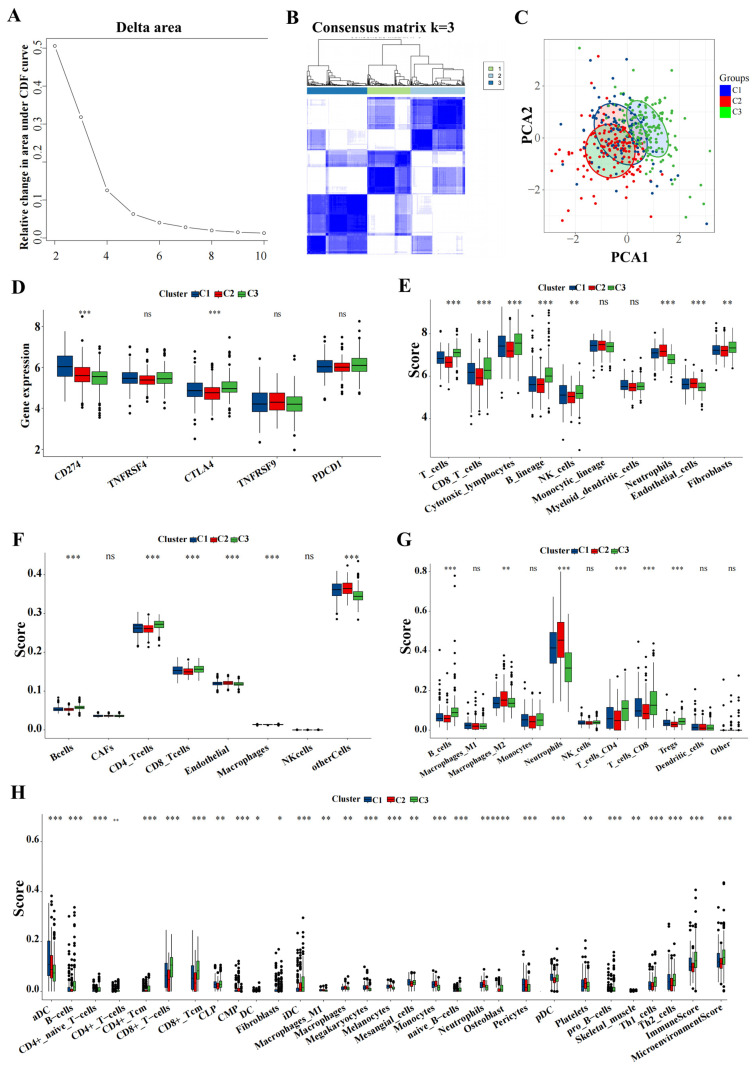
Key genes expression profiling identified three distinct IPF subtypes. (**A**) The CDF curve of the samples and the delta area curve from consensus clustering. The delta area curve represents the degree of variance in the area under the CDF curve for each cluster count (k) compared to (k−1). The horizontal axis depicts the cluster count (k), while the vertical axis shows the relative change in the CDF area. (**B**) Consensus-clustering matrix when (k = 3). (**C**) PCA illustrating significant transcriptomic differences among the three clusters. (**D**–**H**) Analysis of immune cell content differences among the identified subtypes using MCP-counter, CIBERSORT, EPIC, quanTIseq, and xCell. Abbreviations: IPF, Idiopathic pulmonary fibrosis; CDF, cumulative distribution function; PCA, principal component analysis; MCP, microenvironment cell populations-counter; CIBERSORT; EPIC, estimating the proportions of immune and cancer cells. * *p* < 0.05, ** *p* < 0.01, *** *p* < 0.001, and ns means no significance.

Utilizing the delta area and heatmap distribution, IPF was stratified into three distinct ICD subtypes designated C1, C2, and C3 based on the expression patterns of eight model genes (Figure 8A,B). PCA revealed significant differences among these subtypes (Figure 8C). Further investigation into the differences in the expression of immunotherapy targets among the subtypes was conducted. Techniques including MCP, CIBERSORT, EPIC, quanTIseq, and xCell were utilized to quantify the immune cell content in IPF, facilitating a comparative analysis of the variances (Figure 8D–H).

### 3.8. Association between the IPF Subclasses and Metabolism-Associated Signatures

**Figure 9:**
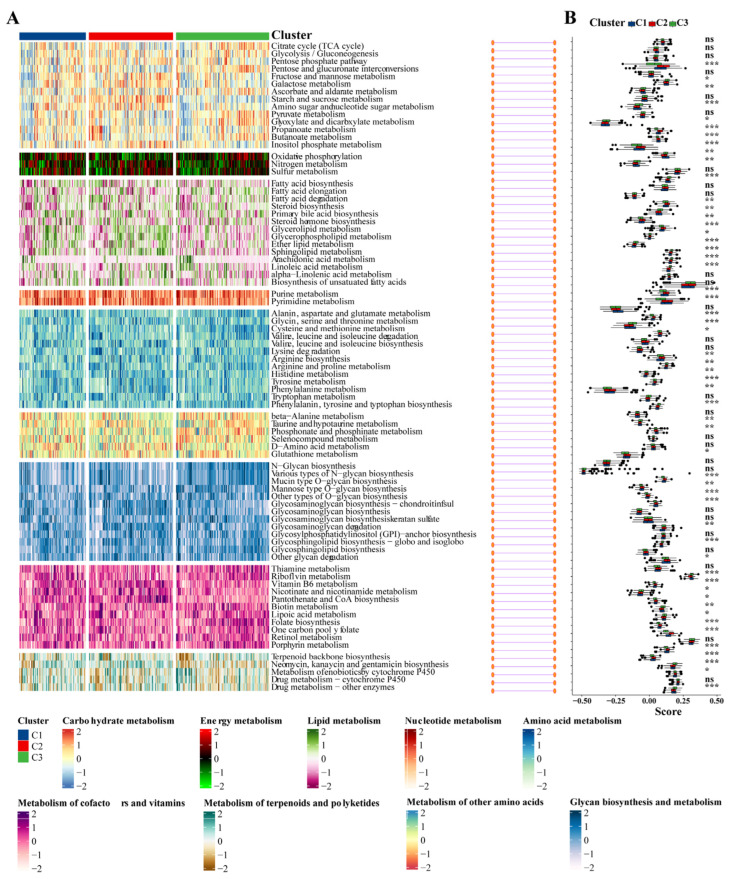
Association between metabolism-associated signatures and the IPF subclasses. (**A**) Heatmap of metabolism-associated signatures. (**B**) Boxplot of the signature score for the metabolism-associated signatures distinguished by different subclasses. Abbreviations: IPF, Idiopathic pulmonary fibrosis. * *p* < 0.05, ** *p* < 0.01, *** *p* < 0.001, and ns means no significance.

Given that previous studies indicated that abnormal metabolic pathways play a critical role in lung fibrosis [31], we investigated whether different subtypes exhibit distinct metabolic characteristics. Initially, 81 metabolic processes were measured, followed by differential analysis to identify subtype-specific metabolic features, characterized by higher GSVA scores in the corresponding subtypes. Indeed, the results demonstrated that the subtypes exhibited different metabolic profiles: the C2 group was mainly associated with energy metabolism and lipid metabolism, while the C3 group was primarily related to the metabolism of cofactors and vitamins (Figure 9A,B).

### 3.9. The rs9265808 Locus in MS4A4A Is a Susceptibility Locus for Progression from Pulmonary Fibrosis to LC

**Figure 10:**
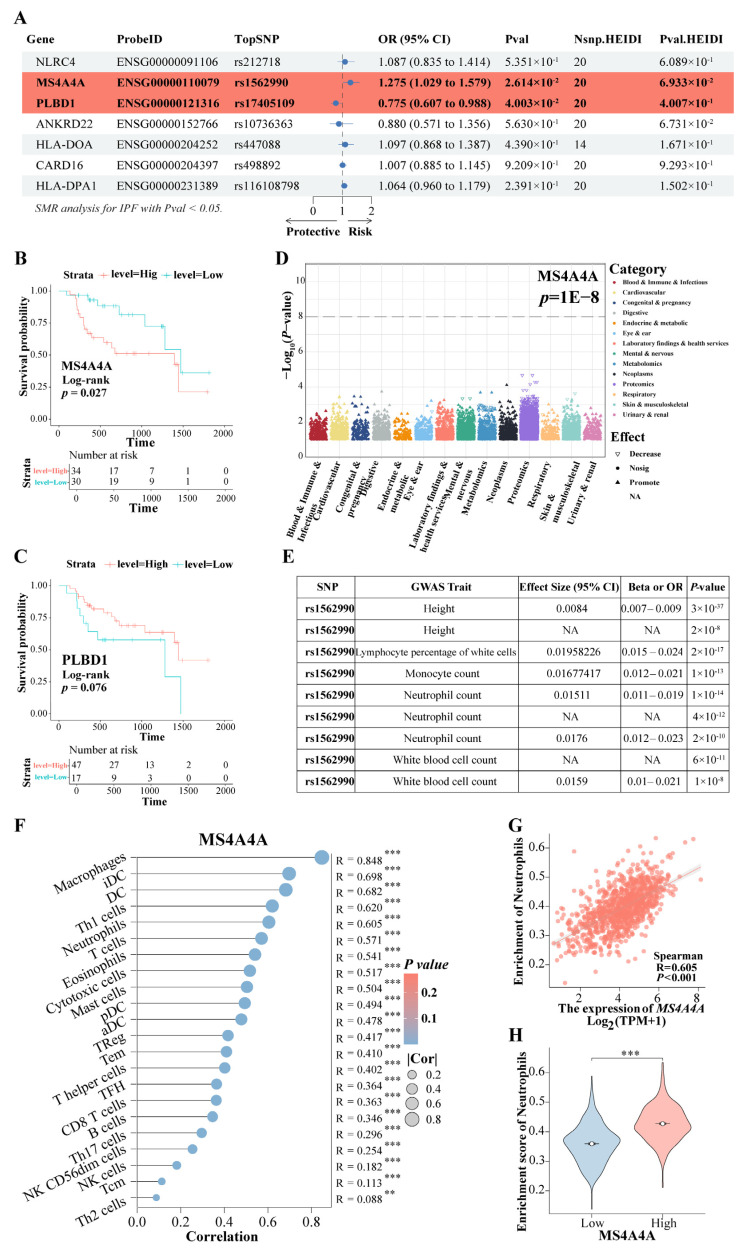
The rs9265808 locus in *MS4A4A* is a susceptibility locus. (**A**) The SMR evaluated the causal relationship between the expression of key genes and IPF risk. (**B**) KM survival analysis of *MS4A4A*. (**C**) KM survival analysis of *PLBD1*. (**D**) PheWAS analyzed the traits associated with *MS4A4A* expression. (**E**) The GWAS trait associated with the most significant rs1562990 locus in *MS4A4A*. (**F**) The correlation between *MS4A4A* and various immune cell. (**G**) The correlation between *MS4A4A* and neutrophils. (**H**) A comparative analysis of neutrophil enrichment scores was conducted among LC patients exhibiting high and low expression of *MS4A4A*. Abbreviations: SMR, summary-data-based Mendelian randomization analysis; IPF, Idiopathic pulmonary fibrosis; KM, Kaplan–Meier; PheWAS, phenome-wide association study. ** represents *p* < 0.01; *** represents *p* < 0.001.

Based on the findings mentioned above, the expression of the eight genes was further evaluated for causal relationships with IPF risk using SMR. The results indicated that the increased expression of the gene *MS4A4A* was associated with a higher risk of IPF (OR = 1.275, 95% CI: 1.029–1.579, *p* = 0.026), whereas the elevated expression of *PLBD1* was found to have a protective effect against IPF (OR = 0.775, 95% CI: 0.607–0.988, *p* = 0.040) (Figure 10A). Both *MS4A4A* and *PLBD1* passed the HEIDI test. Survival analysis demonstrated that IPF patients with high *MS4A4A* expression in lung tissue had significantly shorter survival times compared to those with low expression (*p* = 0.027) (Figure 10B), while no significant difference in survival time was observed for *PLBD1* expression (Figure 10C). PheWAS analysis did not identify any significant traits associated with *MS4A4A* expression (Figure 10D). This finding suggests a low likelihood of false positive causal effects due to pleiotropy revealed via the SMR analysis, indicating feasible safety in targeting *MS4A4A* to prevent the progression of IPF to LC. Among the SNPs, rs1562990 was the most significant for *MS4A4A* and was associated with certain blood routine indices and patient height, particularly with neutrophils, monocytes, and white blood cells (Figure 10E). It has been reported that *MS4A4A* plays a role in lung inflammation by regulating immune cells [32]. Additionally, a strong correlation was found between *MS4A4A* and various immune cell levels in LC (Figure 10F), notably with neutrophils (R = 0.605, *p* < 0.001) (Figure 10G). In LC patients with high *MS4A4A* expression, the neutrophil enrichment score was significantly increased (*p* < 0.001) (Figure 10H). These results suggest that lung fibrosis may reprogram the lung environment to favor neutrophil activity, thereby increasing the risk of LC. The increased expression of *MS4A4A* in neutrophils unexpectedly raises the likelihood of LC development.

### 3.10. MS4A4A Deficiency Alleviates Pulmonary Fibrosis in Mice

**Figure 11:**
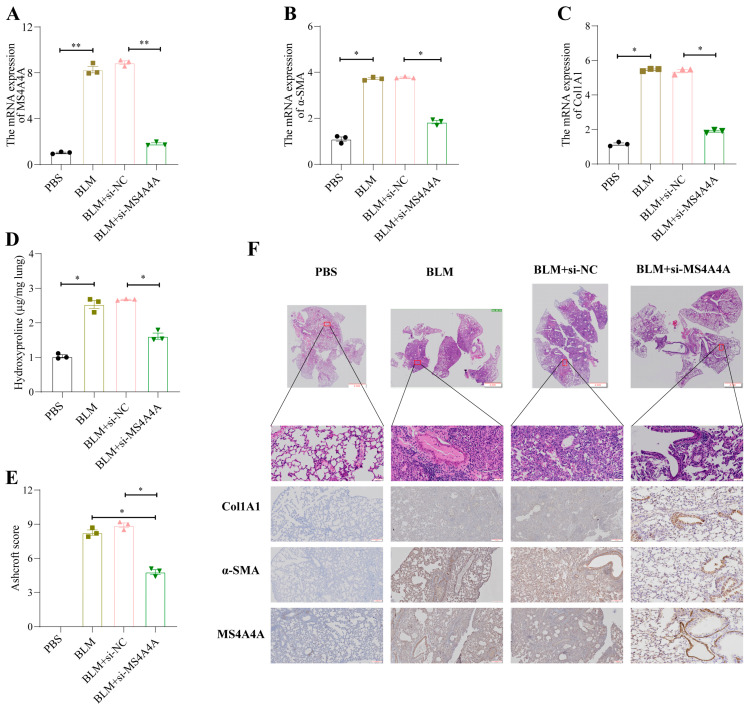
*MS4A4A* was a potential therapeutic target for pulmonary fibrosis. (**A**) Tracheal MS4A4A lentivirus inhalation inhibited the relative expression of *MS4A4A* induced by BLM. (**B**,**C**) Tracheal inhalation of MS4A4A lentivirus inhibited the relative expression of BLM-induced fibrosis-related gene α-*SMA* (**B**) and *Col1A1* (**C**). (**D**) The content of hydroxyproline in lung of *MS4A4A* lentivirus treated mice decreased significantly. (**E**) Ashcroft score analysis for evaluating fibrosis severity in mice from various groups. (**F**) Histological staining (HE and IHC, α-*SMA*, *Col1A1* and *MS4A4A*) demonstrating that treatment with *MS4A4A* mitigates BLE-induced lung morphological alterations and fibrotic area expansion (scale bars: 2 mm, 50 µm and 200 µm). Abbreviations: BLM, bleomycin; HE, Hematoxylin and Eosin staining; IHC, Immunohistochemistry. Statistical significance between groups was determined using two-sided unpaired *t*-tests. * *p* < 0.05, and ** *p* < 0.01. Data are presented as mean ± standard error (SE). Data normality was verified with the Shapiro–Wilk test, and homogeneity of variances was confirmed using Levene’s test, with all data passing these checks.

To validate the preventive effect of *MS4A4A* on pulmonary fibrosis in vivo, an adenovirus-associated virus expressing si-*MS4A4A* was constructed and administered intratracheally to mice on the seventh day following bleomycin (BLM) induction. The preventive efficacy against pulmonary fibrosis was subsequently observed. Mice were euthanized on the twenty-first day, and lung tissues were harvested for further analysis. The qRT-PCR analysis revealed elevated levels of *MS4A4A*, *α-SMA*, and *Col1A1* in the lungs of the BLM-treated mice; however, the administration of si-MS4A4A effectively blocked the BLM-induced upregulation of *α-SMA* and *Col1A1* expression (Figure 11A–C). The hydroxyproline assay results indicated a significant reduction in hydroxyproline levels in the si-*MS4A4A* group, highlighting its therapeutic effect (Figure 11D). The Ashcroft scoring system confirmed that BLM successfully induced severe structural distortion and expanded fibrotic regions in the lungs of the mice, and these effects were mitigated upon the inhalation of si-*MS4A4A* (Figure 11E). HE staining demonstrated appreciable improvements in lung tissue morphology and reduced fibrotic areas in the si-*MS4A4A* group, indicating the in vivo inhibition of fibrosis development (Figure 11F). Immunohistochemistry further revealed that the si-*MS4A4A* treatment significantly suppressed the BLM-induced increase in *Col1A1* and *α-SMA* expression (Figure 11F).

## 4. Discussion

IPF has long been identified as a significant risk factor for LC. The incidence of LC in patients with IPF is significantly greater, with the risk being five times greater than that in the general population, ranging from 3% to 22% [33]. It has been reported that the all-cause mortality rates for IPF patients diagnosed with LC are 53.5%, 78.6%, and 92.9% at 1 year, 3 years, and 5 years, respectively [34]. Genomic profiling by Hwang et al. [35] revealed common mutations in the *TP53* and *BRAF* genes in patients with IPF-LC. Additionally, deletion of the *FHIT* allele has been implicated in the carcinogenesis of peripheral lung tissue in IPF patients [36]. To date, the exact mechanisms linking IPF and LC have yet to be fully elucidated.

In this study, a causal relationship between IPF and LC was identified from the perspective of genetic variation. In individuals with genetic predispositions, recurrent, unexplained injuries trigger fibroblast activation, abnormal extracellular matrix accumulation, and aberrant bronchiolization of the alveolar epithelium. The continuous interaction of these elements fosters the development and progression of LC.

The critical role of the immune response in the etiology of IPF has been increasingly recognized. [37,38]. Every stage of fibrogenesis is accompanied by both innate and adaptive immune responses [37]. Furthermore, ICD plays a significant role in both the pathogenesis of and treatment strategies for pulmonary fibrosis [39,40,41]. For instance, elevated levels of IL-18, which modulates EMT in a Snail-1-dependent manner, have been implicated in the progression of bleomycin-induced pulmonary fibrosis [42]. Studies have demonstrated that regulating the expression of ICD markers can alleviate pulmonary fibrosis in bleomycin-induced models [41,43]. Therapeutic agents targeting ICDs have been shown to be effective in ameliorating fibrogenesis [44].

In pulmonary fibrosis, dysregulated immune responses can result in tissue damage and fibrosis. Differentially expressed genes were identified from a lung cancer dataset and a combined dataset of five IPF studies. WGCNA was conducted to reveal six LC-related gene modules and seven IPF-related gene modules. The MEbrown module in LC patients and the MEblue module in IPF patients were significantly associated with ICD. Cross-referencing these two modules revealed 22 shared genes. Subsequently, eight genes were further screened using four machine-learning methods, and a predictive model was established using MLP, which demonstrated high accuracy upon validation. A nomogram incorporating these eight genes was constructed to assist in the clinical diagnosis of IPF. The nomogram’s accuracy was confirmed via calibration curves, decision curves, and ROC curves in both the training and validation sets. Validation at the single-cell level indicated that these genes could not only aid clinicians in diagnosing IPF but could also potentially serve as therapeutic targets.

Despite the identification of risk genes for IPF, a comprehensive understanding of the genetic basis of the disease remains incomplete. The integration of the GWAS and cis-eQTL datasets was successful in enhancing the discovery of risk genes and providing biological insights into their functions. SMR analysis indicated a causal relationship between the expression of *MS4A4A* and the risk of IPF, with elevated expression levels increasing the disease risk. Additionally, the PheWAS results suggested that targeting *MS4A4A* for IPF treatment is feasible from a safety perspective. It was also found that the increased expression of *MS4A4A* in neutrophils may promote the progression of IPF to LC. Neutrophils have been shown to directly induce the death of epithelial and endothelial cells, leading to lung injury and tumor formation [45]. Therefore, it is hypothesized that MS4A4A facilitates the progression of IPF to LC via neutrophil induction. In vivo animal experiments further supported that inhibiting *MS4A4A* expression mitigated lung fibrosis. *MS4A4A*, a member of the transmembrane 4A protein family [46], is involved in regulating calcium signaling and is considered a marker of M2 macrophages [47]. It has been linked to autoimmune diseases such as rheumatoid arthritis [48], systemic sclerosis [49], and Kawasaki disease [50]. *MS4A4A* plays a role in lung inflammation through the modulation of immune cells [32]. Previous studies have also shown that a high level of *MS4A4A* expression is significantly associated with a poor prognosis in various cancers [51,52]. These findings corroborate our results, implicating *MS4A4A* as a risk factor in both IPF and lung cancer, potentially contributing to lung tissue damage and fibrosis through immune system dysregulation. Furthermore, in vivo experiments demonstrated that inhibiting *MS4A4A* expression ameliorates lung fibrosis, suggesting that *MS4A4A* is a promising therapeutic target.

A variety of analytical methods, including TSMR, SMR, PheWAS, and WGCNA, were utilized in this study to comprehensively explore therapeutic targets in IPF. However, several limitations were identified, such as data noise and uncertainty, predictive models, causal relationships, and an incomplete understanding of biological networks. First, although MR and SMR methods can provide evidence for potential causal relationships, establishing a definitive causal link between IPF and LC onset or progression remains a significant challenge, necessitating further experimental validation. Second, transcriptomic data include gene expression levels within a specific context, potentially overlooking dynamic regulatory mechanisms involved in the development of IPF and LC. Finally, the functionality of the key gene *MS4A4A* and the in vivo efficacy of potential therapeutic agents require additional experimental and clinical research for validation.

## 5. Conclusions

This study confirms a causal relationship between IPF and LC, underscoring IPF’s genetic predisposition to progress to LC. The newly developed prediction model demonstrated a high predictive accuracy for IPF. Additionally, *MS4A4A* emerges as a significant therapeutic target, with its potential to prevent the transition from IPF to LC providing a promising avenue for future interventions.

## Data Availability

The datasets used and/or analyzed in the current study were all obtained through public databases with links included in the manuscript. If there is a further need, it can be provided on reasonable request to the corresponding author.

## Supplementary Materials

The following supporting information can be downloaded at: https://www.mdpi.com/article/10.3390/biomedicines12102382/s1, Figure S1: Funnel plots. Figure S2: Scatter plots. Figure S3: Leave-one-out results. Table S1: GWAS data sources and information.
