# Correction: Xie et al. Exploring the Mechanisms and Preventive Strategies for the Progression from Idiopathic Pulmonary Fibrosis to Lung Cancer: Insights from Transcriptomics and Genetic Factors. *Biomedicines* 2024, *12*, 2382

**DOI:** 10.3390/biomedicines13051096

**Published:** 2025-04-30

**Authors:** Kai Xie, Xiaoyan Tan, Zhe Chen, Yu Yao, Jing Luo, Haitao Ma, Yu Feng, Wei Jiang

**Affiliations:** 1 Department of Thoracic and Cardiovascular Surgery, Medical Center of Soochow University, Suzhou 215000, China; kaixie715786420@126.com (K.X.); 20225256025@stu.suda.edu.cn (X.T.); czstudy1998@163.com (Z.C.); mhtszdx@163.com (H.M.); 2 Department of Respiratory Medicine, Nanjing University of Chinese Medicine, Nanjing 210000, China; yaoyuxixi1993@163.com; 3 Department of Cardiothoracic Surgery, Medical School of Nanjing University, Nanjing 210002, China; luojing_2767983@163.com; 4 Department of The First Clinical, Medical College of Soochow University, Suzhou 215006, China

## Error in Figure

In the original publication, there was a mistake in Figure 11 as published [1]. One of the sub-images was mistakenly used during the assembly process. Additionally, to maintain consistent height and width across the panels, some distortion of the sub-images occurred.

The corrected Figure 11 appears below. The authors state that the scientific conclusions are unaffected. This correction was approved by the Academic Editor. The original publication has also been updated.

## Figures and Tables

**Figure 11:**
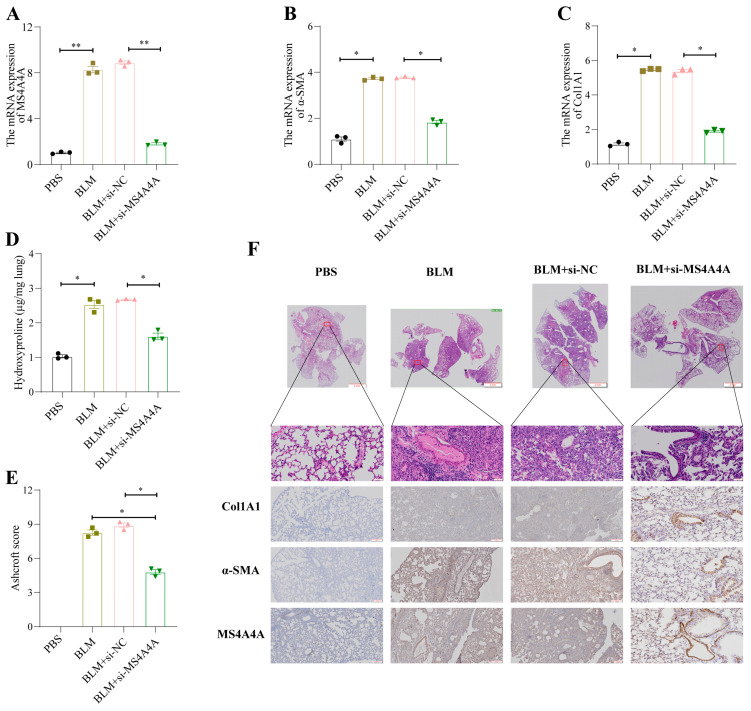
*MS4A4A* was a potential therapeutic target for pulmonary fibrosis. (**A**) Tracheal MS4A4A lentivirus inhalation inhibited the relative expression of *MS4A4A* induced by BLM. (**B**,**C**) Tracheal inhalation of MS4A4A lentivirus inhibited the relative expression of BLM-induced fibrosis-related gene α-*SMA* (**B**) and *Col1A1* (**C**). (**D**) The content of hydroxyproline in lung of *MS4A4A* lentivirus treated mice decreased significantly. (**E**) Ashcroft score analysis for evaluating fibrosis severity in mice from various groups. (**F**) Histological staining (HE and IHC, α-*SMA*, *Col1A1* and *MS4A4A*) demonstrating that treatment with *MS4A4A* mitigates BLE-induced lung morphological alterations and fibrotic area expansion (scale bars: 2 mm, 50 µm and 200 µm). Abbreviations: BLM, bleomycin; HE, Hematoxylin and Eosin staining; IHC, Immunohistochemistry. Statistical significance between groups was determined using two-sided unpaired *t*-tests. * *p* < 0.05, and ** *p* < 0.01. Data are presented as mean ± standard error (SE). Data normality was verified with the Shapiro–Wilk test, and homogeneity of variances was confirmed using Levene’s test, with all data passing these checks.
