# Supplementary material for: Exploring the Mechanisms and Preventive Strategies for the Progression from Idiopathic Pulmonary Fibrosis to Lung Cancer: Insights from Transcriptomics and Genetic Factors
Source: Biomedicines. 2024 Oct 18;12(10):2382. doi: 10.3390/biomedicines12102382 (PMC11504276; doi:10.3390/biomedicines12102382)
Supplement: Supplementary file 1 [file biomedicines-12-02382-s001.zip › Supplementary Figure S1.pdf]

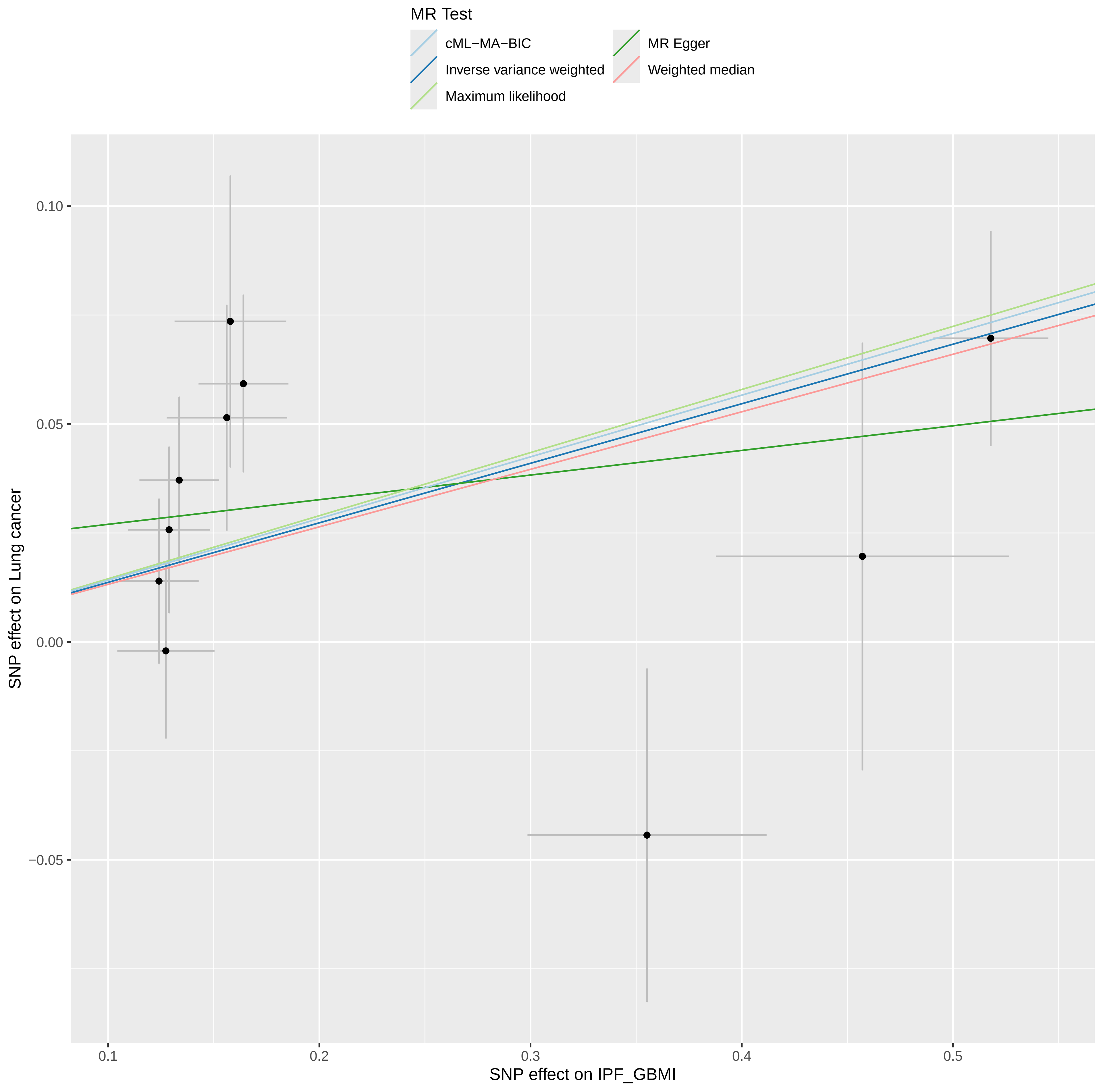

MR Test
cML−MA−BIC
Inverse variance weighted
Maximum likelihood
MR Egger
Weighted median
0.10
0.05
0.00
−0.05
SNP effect on Lung cancer
0.1
0.2
0.3
0.4
0.5
SNP effect on IPF_GBMI

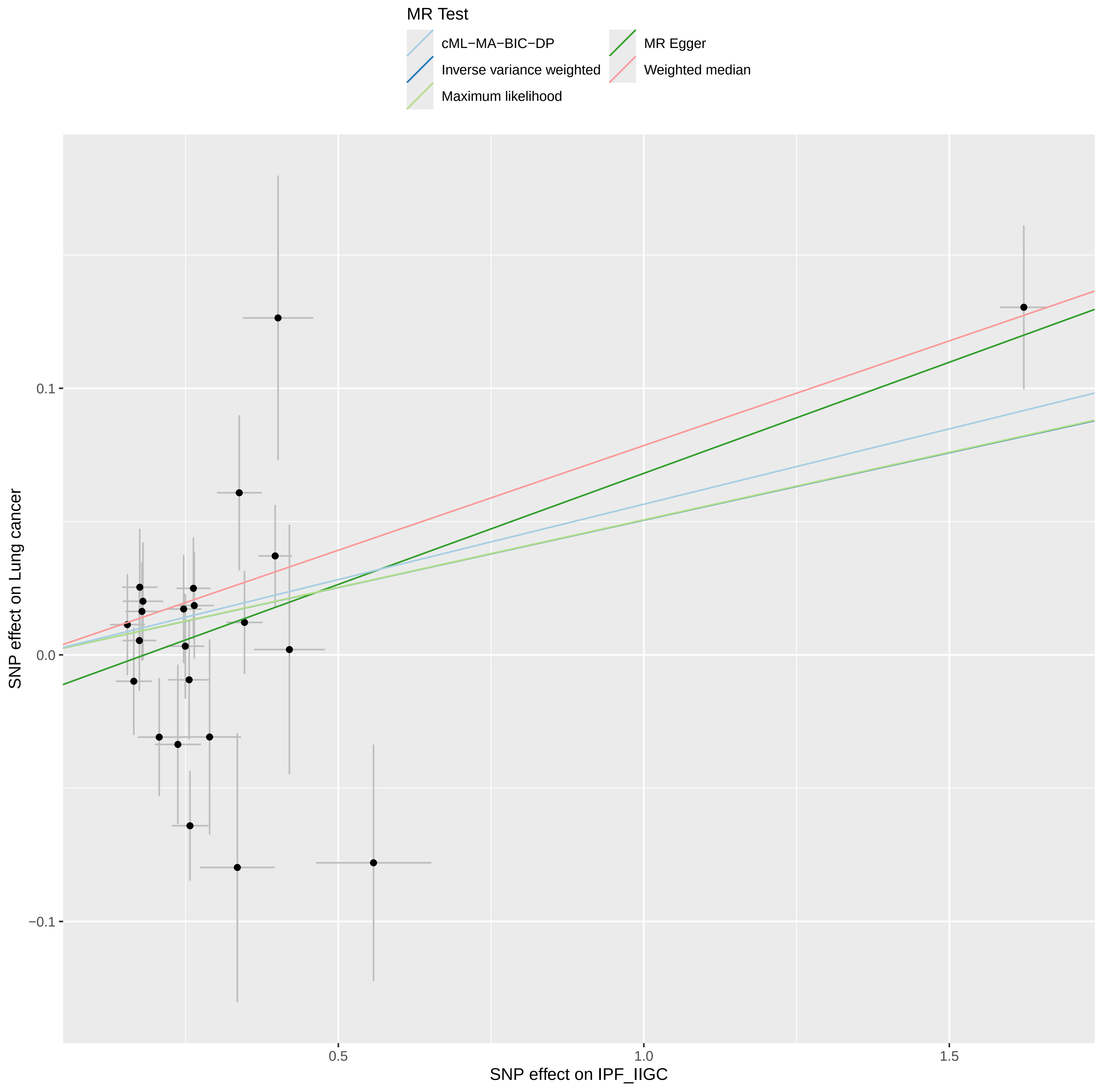

MR Test
cML−MA−BIC−DP
Inverse variance weighted
Maximum likelihood
MR Egger
Weighted median
SNP effect on Lung cancer
0.1
0.0
−0.1
0.5
1.0
1.5
SNP effect on IPF_IIGC
