# Supplementary figures and images for: Exploring the Mechanisms and Preventive Strategies for the Progression from Idiopathic Pulmonary Fibrosis to Lung Cancer: Insights from Transcriptomics and Genetic Factors
Source: Biomedicines. 2024 Oct 18;12(10):2382. doi: 10.3390/biomedicines12102382 (PMC11504276; doi:10.3390/biomedicines12102382)

MR Method

- Inverse variance weighted
- MR Egger

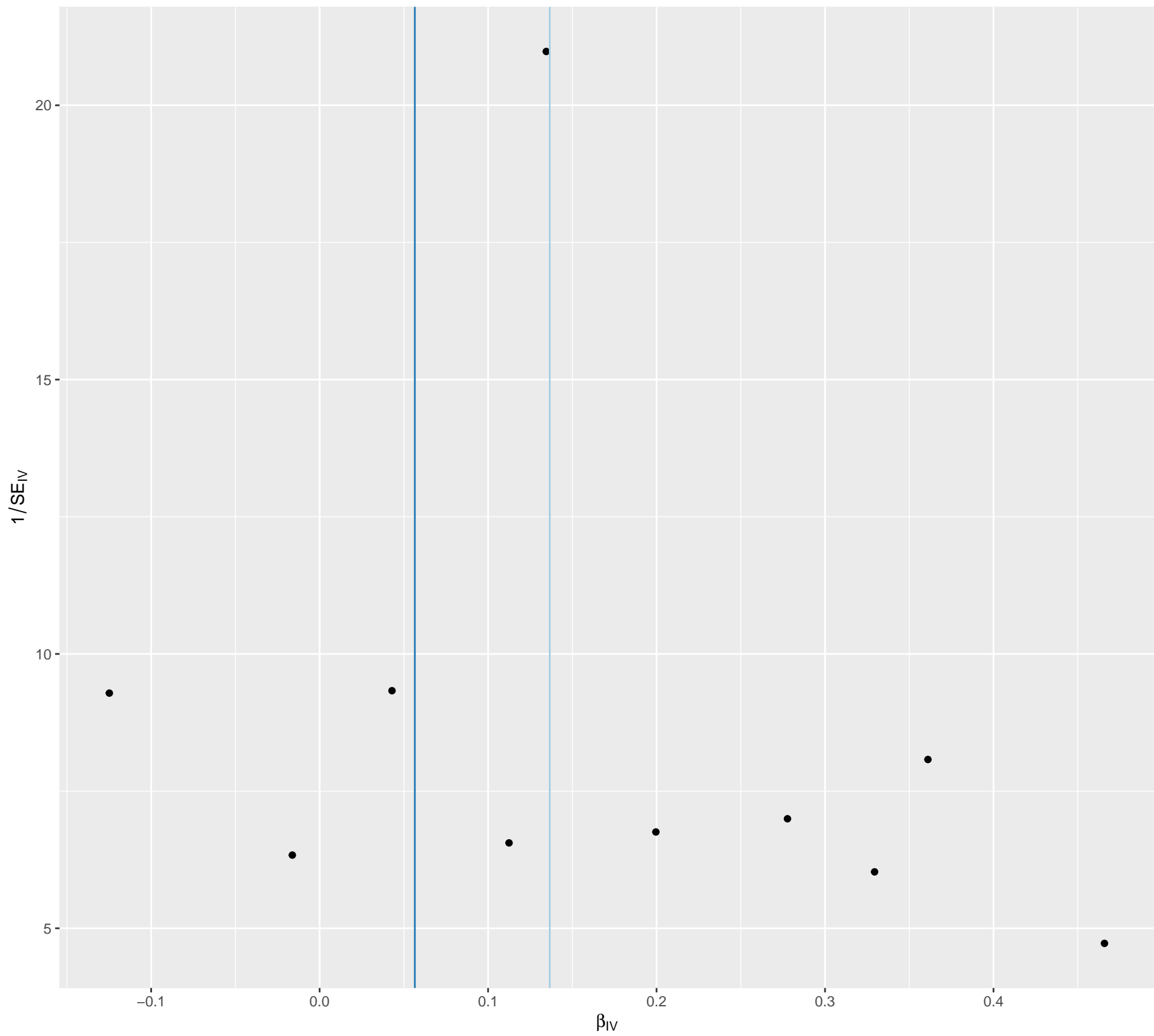

# MR Method

- Inverse variance weighted
- MR Egger

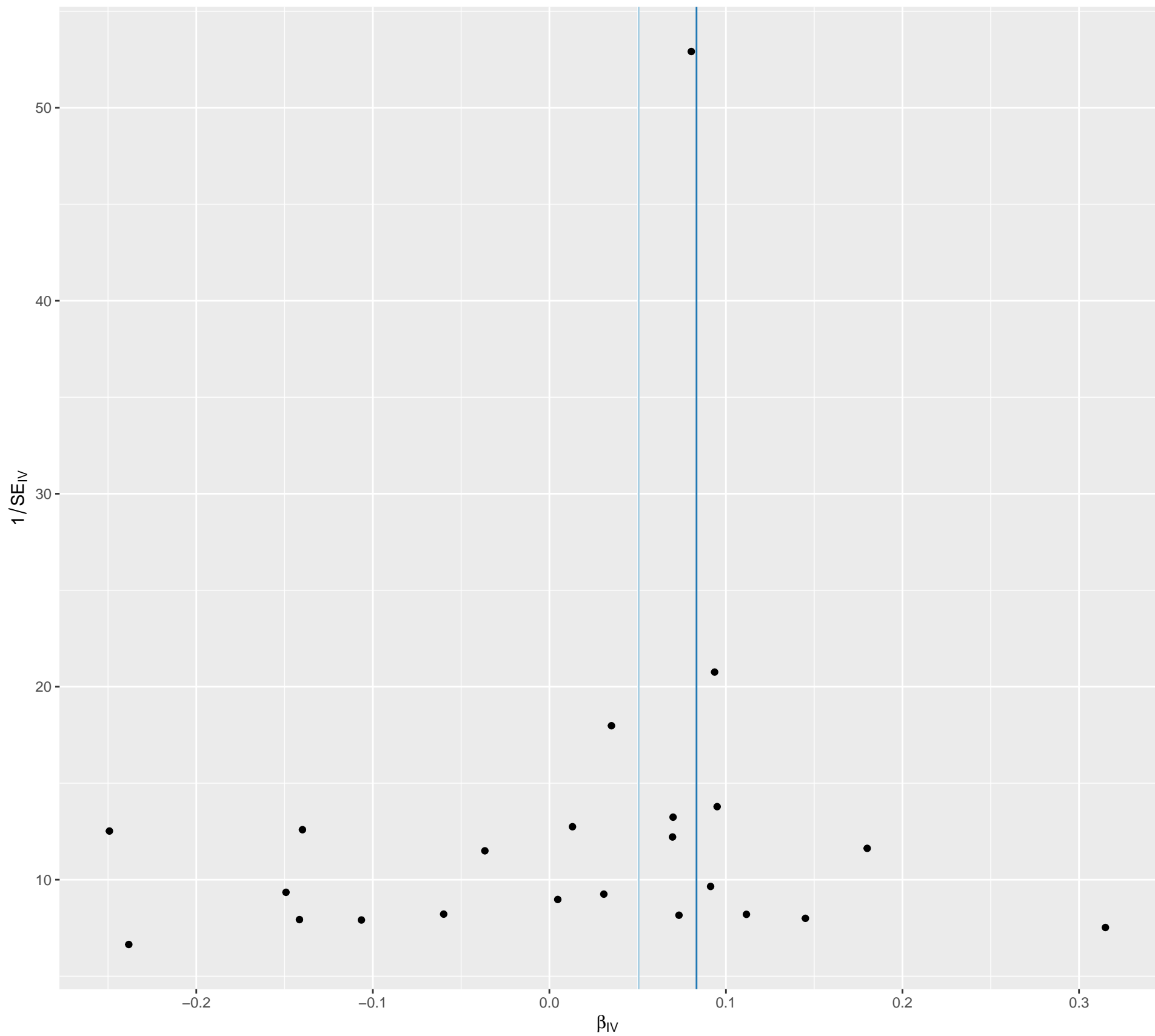

Supplement: Supplementary file 1 [file biomedicines-12-02382-s001.zip › Supplementary Figure S2.pdf]

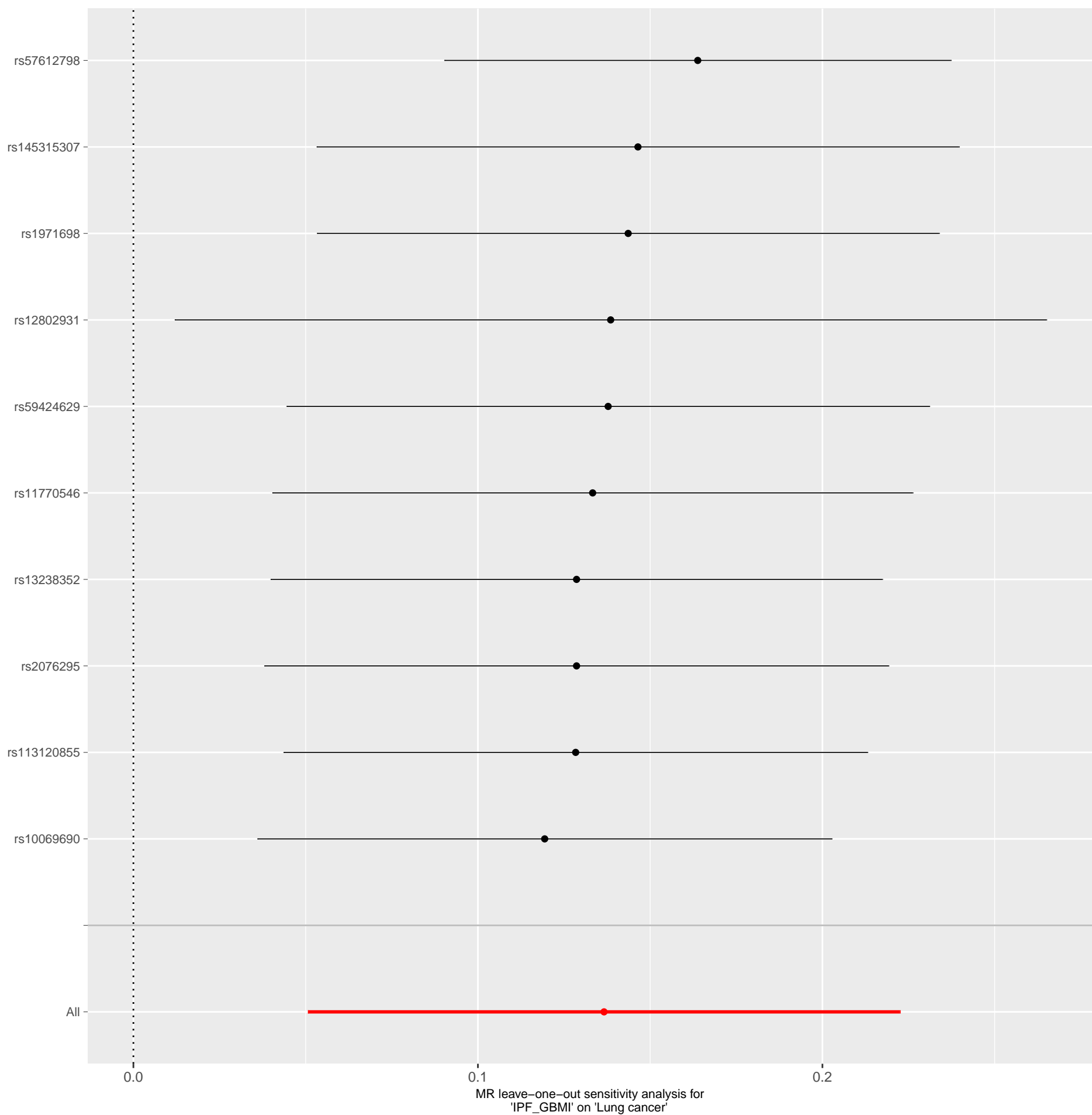

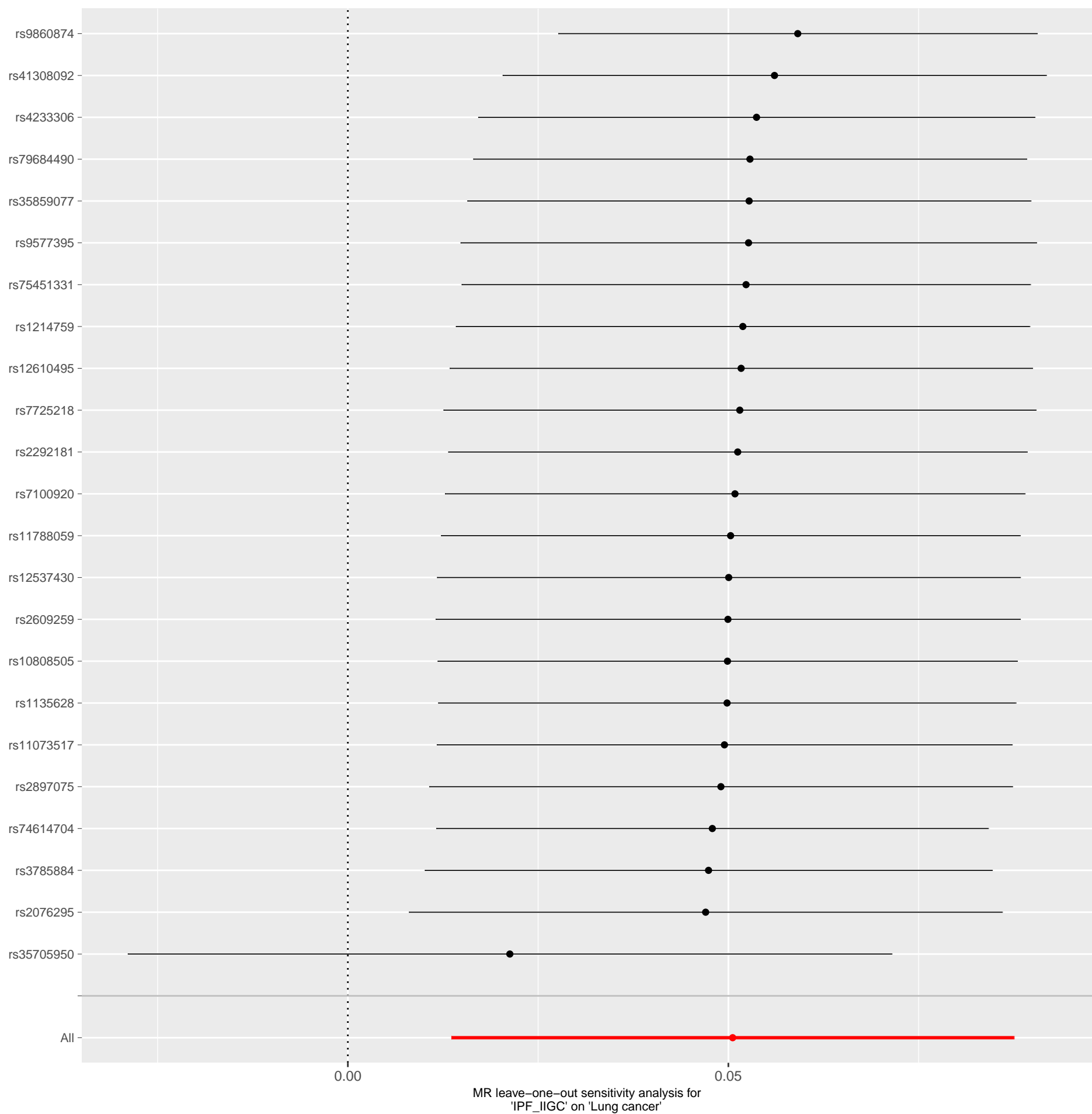

Supplement: Supplementary file 1 [file biomedicines-12-02382-s001.zip › Supplementary Figure S3.pdf]
